# Phytol, a Diterpene Alcohol from Chlorophyll, as a Drug against Neglected Tropical Disease Schistosomiasis Mansoni

**DOI:** 10.1371/journal.pntd.0002617

**Published:** 2014-01-02

**Authors:** Josué de Moraes, Rosimeire N. de Oliveira, Jéssica P. Costa, Antonio L. G. Junior, Damião P. de Sousa, Rivelilson M. Freitas, Silmara M. Allegretti, Pedro L. S. Pinto

**Affiliations:** 1 Faculdade de Ciências de Guarulhos, FACIG, Guarulhos, São Paulo, Brazil; 2 Departamento de Biologia Animal, Instituto de Biologia, Universidade Estadual de Campinas, São Paulo, Brazil; 3 Programa de Pós-graduação em Biotecnologia, Universidade Federal do Piauí, Teresina, Piauí, Brazil; 4 Programa de Pós-graduação em Ciências Farmacêuticas, Universidade Federal do Piauí, Teresina, Piauí, Brazil; 5 Departamento de Fisiologia, Universidade Federal da Paraíba, João Pessoa, Paraíba, Brazil; 6 Núcleo de Enteroparasitas, Instituto Adolfo Lutz, São Paulo, São Paulo, Brazil; McGill University, Canada

## Abstract

**Background:**

Schistosomiasis is a major endemic disease that affects hundreds of millions worldwide. Since the treatment and control of this parasitic disease rely on a single drug, praziquantel, it is imperative that new effective drugs are developed. Here, we report that phytol, a diterpene alcohol from chlorophyll widely used as a food additive and in medicinal fields, possesses promising antischistosomal properties *in vitro* and in a mouse model of schistosomiasis mansoni.

**Methods and findings:**

*In vitro*, phytol reduced the motor activity of worms, caused their death and confocal laser scanning microscopy analysis showed extensive tegumental alterations in a concentration-dependent manner (50 to 100 µg/mL). Additionally, phytol at sublethal doses (25 µg/mL) reduced the number of *Schistosoma mansoni* eggs. *In vivo*, a single dose of phytol (40 mg/kg) administered orally to mice infected with adult *S. mansoni* resulted in total and female worm burden reductions of 51.2% and 70.3%, respectively. Moreover, phytol reduced the number of eggs in faeces (76.6%) and the frequency of immature eggs (oogram pattern) was significantly reduced. The oogram also showed increases in the proportion of dead eggs. Confocal microcopy studies revealed tegumental damage in adult *S. mansoni* recovered from mice, especially in female worms.

**Conclusions:**

The significant reduction in parasite burden by this chlorophyll molecule validates phytol as a promising drug and offers the potential of a new direction for chemotherapy of human schistosomiasis. Phytol is a common food additive and nonmutagenic, with satisfactory safety. Thus, phytol has potential as a safe and cost-effective addition to antischistosomal therapy.

## Author Summary

Schistosomiasis is an infectious parasitic disease caused by helminths from the genus *Schistosoma*, which affects hundreds of millions of people, mainly the poor. Despite schistosomiasis being one of the most prevalent and debilitating neglected tropical diseases, the treatment and control of this disease relies on a single drug, praziquantel. However, there is increasing concern about the development of drug resistance. For this reason, the search for new schistosomicidal agents is a priority. Here, we report that phytol, a molecule from chlorophyll widely used as a food additive and in medicinal fields, shows promising antischistosomal properties against adult *Schistosoma mansoni in vitro* and in laboratory studies with mice harbouring adult *S. mansoni*. Phytol is a common food additive and is nonmutagenic, with satisfactory safety. Thus, phytol has potential as a safe and cost-effective addition to antischistosomal therapy. Further studies are needed because our results might have public health relevance.

## Introduction

Schistosomiasis still constitutes one of the biggest health problems in the world. This disease of poverty has proved difficult to control for centuries and consequently, it still affects hundreds of millions of people. Recent articles document infection of approximately 200 million people in more than 70 developing countries, with approximately 800 million, mostly children at risk of infection [1]. Additionally, the disease burden is estimated to exceed 70 million disability-adjusted life-years [2]. The causative agents of schistosomiasis are parasitic flatworms of the genus *Schistosoma*. Three species (*Schistosoma mansoni*, *Schistosoma haematobium* and *Schistosoma japonicum*) account for the majority of human infections. The major aetiological agent of human schistosomiasis is *S. mansoni* and intestinal schistosomiasis caused by this species is present in Africa, the Middle East, the Caribbean, and South America. Typically, the morbidity associated with schistosomiasis results from the immunological reactions launched in response to parasite egg deposition in the liver and other host tissues [3].

Despite the public health importance of schistosomiasis and the risk that the disease might further spread and intensify, schistosomiasis control programmes are based are based mainly on chemotherapy, which is limited to the anthelmintic drug praziquantel [4]. However, due to the widespread and intensive use of praziquantel, there is increasing concern about the development of drug-resistant strains [5], [6]. For this reason, the search for new schistosomicidal agents is a priority.

Plants have always been used as a common source of medicine, both for traditional remedies and in industrialised products [7], [8]. Chlorophylls, found in all green vegetables, constitute an important source of an isoprenoid component, phytol (3, 7, 11, 15-tetramethyl-2-hexadecen-1-ol) [9]. It is an acyclic monounsaturated diterpene alcohol, present in vitamin K, vitamin E, and other tocopherols. Phytol is an aromatic ingredient used in many fragrance compounds and it may be found in cosmetic and non-cosmetic products [10]. In medicinal fields, phytol has shown antinociceptive and antioxidant activities [11] as well as anti-inflammatory and antiallergic effects [12]. Recent studies have revealed that phytol is an excellent immunostimulant, superior to a number of commercial adjuvants in terms of long-term memory induction and activation of both innate and acquired immunity [13]. Additionally, phytol and its derivatives have no cumulative inflammatory or toxic effects even in immuno-compromised mice [14]. Phytol has also shown antimicrobial activity against *Mycobacterium tuberculosis* [15], [16] and *Staphylococcus aureus* [17].

Drugs of natural origin have already been used to treat parasitic diseases. In this regard, the search for antischistosomal compounds from natural sources, mainly from plants, has been intensified [18], [19]. We have been specifically interested in phytol since it has well-characterised mechanisms of toxicity, is structurally simple, easily available, and cost-effective. Additionally, phytol is a common food additive and, thus, should be well tolerated by the body [9], [10], [14].

In this paper, we describe the *in vitro* and *in vivo* schistosomicidal activity of phytol against *Schistosoma mansoni* for the first time. As a benchmark, praziquantel was also used *in vitro*. As a first step, *in vitro* antischistosomal studies were performed. Subsequently, a trial was designed to test the schistosomicidal activity of phytol in experimental schistosomiasis caused by *S. mansoni* in a mouse model. We also demonstrated and described the ability of phytol to induce severe membrane damage in schistosomes through the use of confocal laser scanning microscopy. Furthermore, the effects of phytol on pairing and egg production by adult worms were also examined.

## Materials and Methods

### 1. Drugs

**Figure 1:**
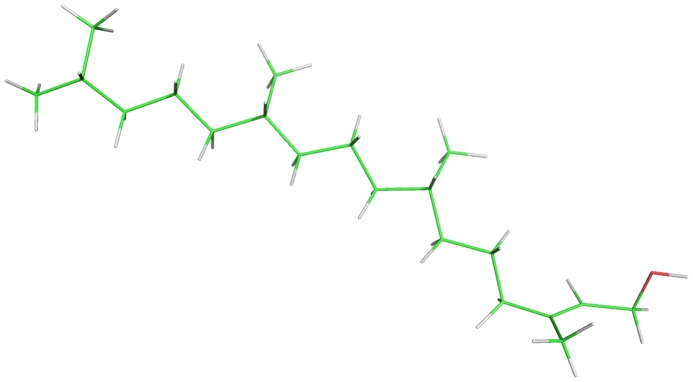
Chemical structure of phytol (3,7,11,15-tetramethylhexadec-2-en-1-ol).

Phytol (**Fig. 1**) was purchased from Sigma-Aldrich (St. Louis, MO, USA) and praziquantel tablets were purchased from Merck (São Paulo, SP, Brazil). For *in vitro* studies, drugs were dissolved in dimethyl sulfoxide (DMSO, Sigma-Aldrich) to obtain stock solutions of 4 mg/mL. For *in vivo* studies, phytol was suspended in 3.7 mL of phosphate buffered saline (PBS) and orally administered at final concentration of 40 mg/kg.

### 2. Animals and parasite

The Belo Horizonte strain of *Schistosoma mansoni* was used in all experiments. The parasite life-cycle is maintained in the laboratory by routine passage through a rodent host and intermediate snail host *Biomphalaria glabrata* [19]. Infections of rodent host with *S. mansoni* were initiated by subcutaneous injection of approximately 150 cercariae. Cercariae were harvested from infected snails by exposure to light for 3 h, following standard procedures of our laboratory [19].

For *in vivo studies*, 3-week-old Balb/c mice were used. Mice were infected with 70 cercariae of *S. mansoni* by tail immersion and kept under environmentally controlled conditions (temperature, 25°C; humidity, 70%) with free access to water and rodent diet [20].

### 3. Ethics statement

The present study was approved by the Ethics Committee at Universidade Federal do Piauí, PI, Brazil (approval number 013/11) and Universidade Estadual de Campinas, SP, Brazil (approval number 2753-1). All the animals were handled in strict accordance with good animal practice as defined by the Universidade Federal do Piauí and Universidade Estadual de Campinas guidelines for animal husbandry, according to with the Brazilian legislation (Comissão de Ética de Uso de Animais, CEUA, 11,794/2008).

### 4. *In vitro* antischistosomal assay

#### a. Parasite preparations and assessment of drug effects

*Schistosoma mansoni* adult worms were obtained by perfusion of hamsters [21], 8 weeks after infection with cercariae. For schistosome preparations and culture, the worm pairs were washed carefully in RPMI 1640 medium kept at pH 7.5 with HEPES 20 mM, supplemented with 10% foetal bovine serum (Gibco BRL) and containing 200 IU/mL penicillin and 200 µg/mL streptomycin (Invitrogen, São Paulo, SP, Brazil). For the *in vitro* test with *S. mansoni*, after washing, schistosomes were incubated in a 24-well culture plate (TPP, St. Louis, MO, USA), placing one coupled worm pair in each well, containing the same medium at 37°C in a 5% CO_2_ atmosphere as previously described [22]–[24]. Phytol was used to obtain final test concentrations of 12.5 to 100 µg/mL (12.5, 25, 50, 75 and 100 µg/mL; equivalent to 42.1, 84.3, 168.6, 252.9 and 337.2 µM) in culture plates with a final volume of 2 mL. The parasites were kept for 120 h and monitored every 24 h using an inverted microscope and ×2.5 and ×10 and objective lens. The effect of the drug was assessed with emphasis on changes in worm motor activity and death of worms as previously described [23]. Changes in the pairing and egg production were also evaluated using an inverted microscope [19], [24], [25]. In addition, worms were prepared for confocal laser scanning microscopy examination whose details are described below. All experiments were carried out in triplicate or quadruplicate and were repeated at least three times. The control worms were assayed in RPMI 1640 medium as negative control group and 1 µg/mL (3.2 µM) praziquantel as positive control group.

#### b. Assessment of the reproductive fitness of adult worms

To evaluate the sexual fitness of worms, the parasites were continually monitored when treated with sub-lethal concentrations of the phytol (3.125, 6.25, and 12.5 µg/mL; equivalent to 10.5, 21.1 and 42.1 µM). Schistosome egg output *in vitro* was determined by counting the number of eggs, as established in our previous works [22], [24]–[26]. *In vitro* egg output was monitored on daily basis for five days using an inverted microscope (Nikon, Japan).

To observe the reversible effect of phytol on the egg output, the medium containing the drug (12.5 µg/mL) was removed after 48 h of drug exposure and the worms were carefully rinsed three times with RPMI to prevent separation of the worm pairs. The worms were then incubated continuously in medium without drug and monitored on daily basis for 72 h [24], [25]. These tests were repeated three times.

### 5. *In vivo* drug treatment

#### a. Infection of mice and experimental design

Mice were infected with approximately 70 *S. mansoni* cercariae by tail immersion. Eight weeks post-infection, groups of ten mice were treated orally with the test drugs using single oral doses of 40 mg of compounds per kg body weight. Control mice were orally administered an equal volume of PBS. Therefore, animals were divided into 2 main groups: infected untreated mice served as controls (Group I, n = 10), and phytol-treated mice (Group II, n = 10). Animals of both groups were sacrificed 2 weeks post-treatment.

*In vivo* experiments with *S. mansoni* were carried out in duplicate. The results from the second set of experiments are summarized in Supporting **Figure S1**. In this case, 7 weeks post-infection, groups of five mice were treated orally with phytol (40 mg/kg) and groups of five untreated mice served as controls.

No data on the potential antischistosomal activity of phytol are available from pilot studies. The decision on the dose to be used was thus primarily guided by experiments describing the activities of praziquantel in humans [27].

In a further attempt to show whether phytol can cause the morphological alterations in adult *Schistosoma mansoni* recovered from mice, a third group of animals was infected and treated as described above. Two days post-treatment, worms were recovered from infected untreated controls (Group IIIa, n = 3) and a phytol-treated group (Group IIIb, n = 3).

#### b. Worm recovery

All groups were killed by cervical dislocation and dissected. Schistosomes were then removed from the hepatic portal system by perfusion technique described above, sexed, and counted [21]. Worms collected from all groups were prepared for examination by confocal laser scanning microscopy. Worm burdens of treated mice were compared to untreated animals and reductions of worm burden calculated.

#### c. Egg developmental stages (oogram pattern)

The assessment of therapeutical efficacy was also based on the quantitative and qualitative oograms technique following criteria previously described [28]. After the perfusion portal system, a fragment of the intestine (10 mm) was cut off and processed for oogram. Eggs were then counted and classified according to different stages of development [28]. They were scored as immature, mature or dead.

#### d. Egg counting in faeces

Kato-Katz method was used for quantitative faeces examination [29]. Faeces samples were collected from all mice, in the two experimental groups, and examined to confront their results with the oogram findings. The slides were examined for *S. mansoni* eggs under a light microscope.

### 6. Confocal laser scanning microscopy studies

To observe morphological changes in the tegument of adult parasites after *in vitro* and *in vivo* assays, schistosomes were monitored using a confocal laser scanning microscope following standard procedures presented elsewhere [19]. Briefly, at the end of the drug treatment period (120 h) or in the case of death, the parasites were fixed in a formalin-acetic acid-alcohol solution (FAA) and analysed under a confocal microscope (Laser Scanning Microscope, LSM 510 META, Carl Zeiss, Standorf Göttingen, Vertrieb, Germany). Autofluorescence was excited with a 488-nm line from an Argon laser, and emitted light was collected with 505 nm [30], [31].

For assessment of changes in the tegument of parasites, three-dimensional images obtained from confocal laser microscopy were used for a quantitative method. In this quantitative analysis, areas of the tegument of male worms are assessed, and the numbers of tubercles were counted according to standard procedures [19]. Briefly, during the microscopic analysis of the three-dimensional images captured using LSM Image Browser software (Zeiss), areas of the tegument of parasite are assessed, and the numbers of intact tubercles on the dorsal surface of male helminths were counted in a 20,000 µm^2^ area.

### 7. Statistical analysis

Statistical tests were performed with GRAPHPAD PRISM (version 5.0) software. Dunnet's test was used to analyze the statistical significance of differences between mean experimental and control values. Significant differences were also determined by applying Tukey's test for multiple comparisons. A *P* value of <0.05 was considered significant.

## Results

### 1. *In vitro* studies

Adult *S. mansoni* worms (56-day-old) were cultured in RPMI 1640 medium in the presence of phytol. The parasites were maintained for 120 h and monitored every 24 h to evaluate their general condition: motor activity, changes in pairing, egg production, alteration in the tegument, and mortality rate.

#### a. Phytol exhibit antischistosomal properties

The results of the *in vitro* studies with 56-day-old adult *S. mansoni* exposed to phytol at concentrations of 12.5, 25, 50, 75 and 100 µg/mL (42.1, 84.3, 168.6, 252.9 and 337.2 µM) and control groups are summarised in **Table 1**.

**Table 1:** *In vitro* effects of phytol against 56-day-old adult *Schistosoma mansoni*.

Group	Period of incubation (h)	Number of worms investigated		Number of dead worms		Motor activity reduction				Worms with tegumental alterations			
						Slight		Significant		Partial		Extensive	
		M	F	M	F	M	F	M	F	M	F	M	F
Control^a^	24	10*	10*	0	0	0	0	0	0	0	0	0	0
	48	10	10	0	0	0	0	0	0	0	0	0	0
	72	10	10	0	0	0	0	0	0	0	0	0	0
	96	10	10	0	0	0	0	0	0	0	0	0	0
	120	10	10	0	0	0	0	0	0	0	0	0	0
0.5% DMSO	24	10	10	0	0	0	0	0	0	0	0	0	0
	48	10	10	0	0	0	0	0	0	0	0	0	0
	72	10	10	0	0	0	0	0	0	0	0	0	0
	96	10	10	0	0	0	0	0	0	0	0	0	0
	120	10	10	0	0	0	0	0	0	0	0	0	0
Praziquantel	24	10	10	10	10	0	0	10	10	0	0	10	10
1 µg/mL	48	10	10	10	10	0	0	10	10	0	0	10	10
(3.2 µM)	72	10	10	10	10	0	0	10	10	0	0	10	10
	96	10	10	10	10	0	0	10	10	0	0	10	10
	120	10	10	10	10	0	0	10	10	0	0	10	10
Phytol	24	10	10	0	0	0	0	0	0	0	0	0	0
12.5 µg/mL	48	10	10	0	0	0	0	0	0	0	0	0	0
(42.1 µM)	72	10	10	0	0	0	0	0	0	0	0	0	0
	96	10	10	0	0	0	0	0	0	0	0	0	0
	120	10	10	0	0	0	0	0	0	0	0	0	0
Phytol	24	10	10	0	0	0	0	0	0	0	0	0	0
25 µg/mL	48	10	10	0	0	0	0	0	0	0	0	0	0
(84.3 µM)	72	10	10	0	0	0	10	0	0	0	10	0	0
	96	10	10	0	0	4	10	0	0	10	10	0	0
	120	10	10	0	0	10	0	0	10	10	0	10	0
Phytol	24	10	10	0	10	0	0	0	10	0	10	0	10
50 µg/mL	48	10	10	0	10	0	10	0	10	0	10	0	10
(168.6 µM)	72	10	10	0	10	4	0	6	10	10	0	0	10
	96	10	10	10	10	0	0	10	10	0	0	10	10
	120	10	10	10	10	0	0	10	10	0	0	10	10
Phytol	24	10	10	0	10	0	0	0	10	0	0	0	10
75 µg/mL	48	10	10	0	10	10	0	0	10	10	0	0	10
(252.9 µM)	72	10	10	10	10	0	0	10	10	0	0	10	10
	96	10	10	10	10	0	0	10	10	0	0	10	10
	120	10	10	10	10	0	0	10	10	0	0	10	10
Phytol	24	10	10	10	10	0	0	10	10	0	0	10	10
100 µg/mL	48	10	10	10	10	0	0	10	10	0	0	10	10
(337.2 µM)	72	10	10	10	10	0	0	10	10	0	0	10	10
	96	10	10	10	10	0	0	10	10	0	0	10	10
	120	10	10	10	10	0	0	10	10	0	0	10	10

a RPMI 1640.

* Values correspond to the sum of the adult schistosomes obtained from three separate experiments performed in triplicate (n = 2) and quadruplicate (n = 1). Male parasite (M). Female parasite (F).

In the negative control groups, when male and female worms were maintained in RPMI 1640 medium containing 0.5% DMSO for 120 h, their appearance was similar to those maintained in the same medium without DMSO. During this period, all male worms attached on the well wall with their ventral sucker and revealed natural peristalsis of the worm body. Usually, female worms were paired with males. On the other hand, 1 µg/mL praziquantel, the positive control, resulted in complete loss of motor activity in all worms and caused the death of all parasites within 24 h without separation of the worms.

The addition of phytol to the culture medium resulted in slightly or significantly reduced motor activity in *S. mansoni*. After adult schistosomes were exposed to phytol at a concentration of 12.5 µg/mL, they showed normal motor activity, and attached their ventral sucker on the wall of the well during the 120 h of incubation. Interestingly, when tested at concentrations of 25 µg/mL and above, all of the adult worm pairs were separated into individual male and female worms after 24 h of incubation. When the concentration of phytol was increased to 50 µg/mL, all *S. mansoni* females showed reduced motor activity and died after 24 h, whereas no mortality was observed in the male worms. However, 100 µg/mL phytol caused the death of 100% of male and female adult parasites within 24 h (**Table 1**).

#### b. Phytol induced severe tegumental damage in schistosomes

Light microscopic investigations demonstrated that phytol at 25 to 100 µg/mL (84.3 to 337.2 µM) caused morphological alterations in the tegument of adult schistosomes (**Table 1**), especially extensive tegumental disruption such as sloughing, with females more susceptible to phytol than males. No tegumental changes in adult worms were observed for the negative control groups (RPMI or RPMI with 0.5% DMSO), while the positive control (1 µg/mL praziquantel) had tegumental alteration in 100% of the worms.

**Figure 2:**
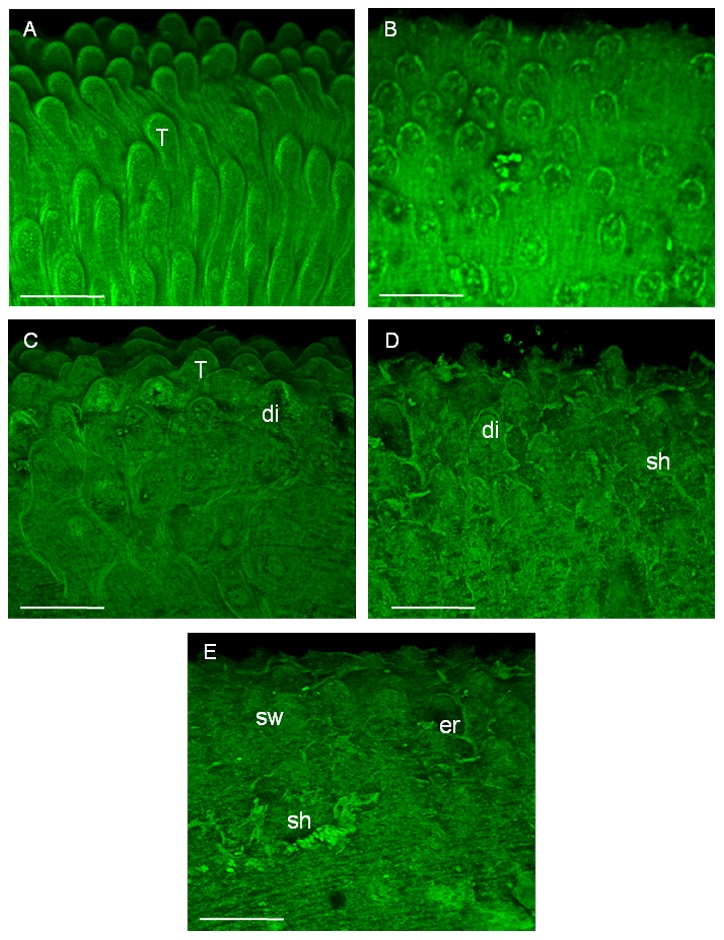
Microscopy investigation of *Schistosoma mansoni* male worm after *in vitro* incubation with phytol. In these experiments, pairs of adult worms were incubated in 24-well culture plates containing RPMI 1640 medium and treated with different concentrations of phytol. After 120 h of incubation or in the case of death, adult male worms were fixed in FAA solution and the fluorescent images were obtained using confocal laser scanning microscopy. **A:** Negative control (RPMI 1640 medium) after 120 h; dorsal tegumental surface showing tubercles (T). **B:** Positive control (1 µg/mL praziquantel) after 24 h. **C:** worm treated with 50 µg/mL of phytol after 96 h; tubercles (T) and the lateral tegument disintegrate (di). **D:** worm treated with 75 µg/mL phytol after 72 h; dorsal tegumental surface showing sloughing (sh). **E:** worm treated with 100 µg/mL phytol after 24 h; dorsal tegumental surface showing swollen (sw), sloughing (sh) and erosion (er). Scale bars = 50 µm.

To further describe the effect of phytol on the tegument of *S. mansoni*, we performed an analysis using confocal laser scanning microscopy. As shown in **Fig. 2**, morphological alterations on the surface of male schistosomes were detected with phytol at concentrations of 50 to 100 µg/mL. No abnormality was seen in the parasites belonging to the negative control group after 120 h (**Fig. 2A**), whereas the treatment in the positive control group (1 µg/mL praziquantel) led to pronounced changes in the tegument of schistosomes after 24 h (**Fig. 2B**). On the other hand, moderate tegumental disruption was observed in the worms exposed to phytol at a concentration of 50 µg/mL after 96 h (**Fig. 2C**), whereas severely damaged worms were seen at higher concentrations (75 and 100 µg/mL after 72 h and 24 h, respectively) (**Fig. 2D** and **Fig. 2E**). In general, the dorsal tegumental surface of male worms showed tubercles that were disintegrated, resulting in the disappearance of knobs as well as swollen, sloughing, and erosion of the surface.

**Figure 3:**
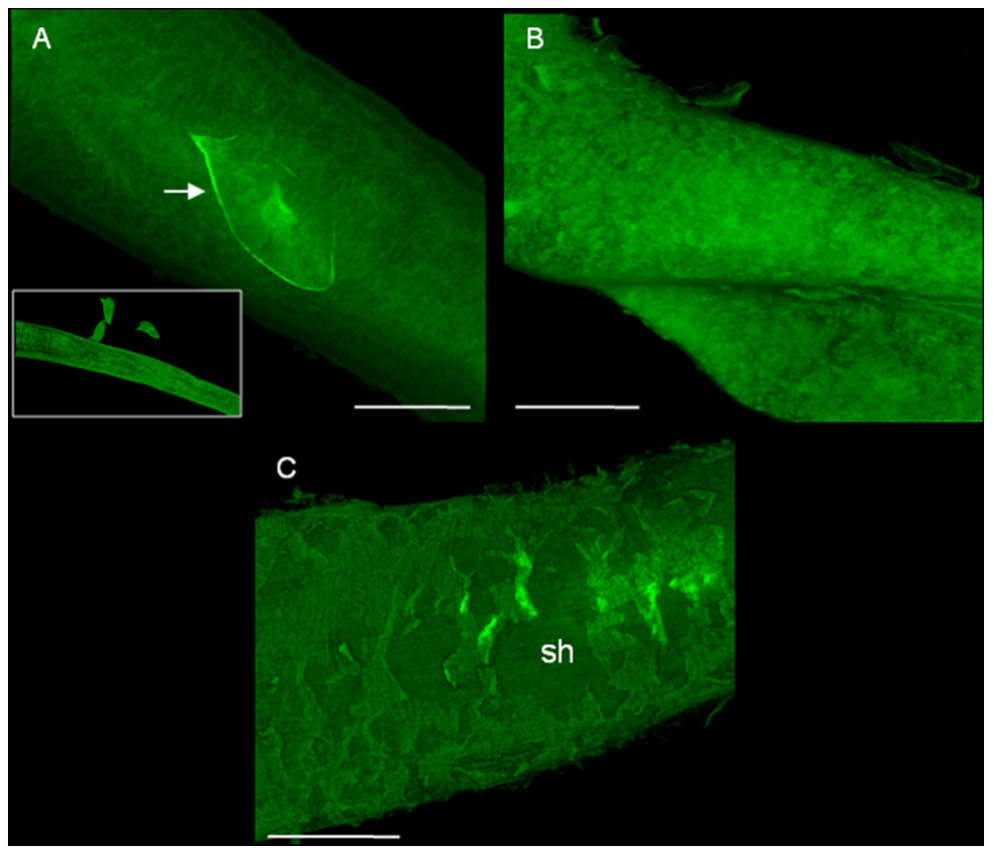
Microscopy investigation of *Schistosoma mansoni* female worm after *in vitro* incubation with phytol. In these experiments, pairs of adult worms were incubated in 24-well culture plates containing RPMI 1640 medium and treated with different concentrations of phytol. After 24 h of incubation or in the case of death, adult female worms were fixed in FAA solution and the fluorescent images were obtained using confocal laser scanning microscopy. **A:** Negative control (RPMI 1640 medium) after 120 h. Female schistosome showing an intra-uterine egg (arrow) and eggs laid by worms are seen (inset). **B:** Positive control (1 µg/mL praziquantel) after 24 h. **C:** Worm treated with 50 µg/mL phytol after 24 h; tegumental surface showing extensive sloughing (sh). Scale bars = 50 µm.

With respect to female schistosomes, confocal microscopic investigations demonstrated that phytol caused morphological alterations in the tegument, especially extensive sloughing after 24 h (**Fig. 3C**). No tegumental changes in adult worms were observed for the negative control group after 120 h (**Fig. 3A**), while the positive control (1.5 µg/mL praziquantel) had tegumental alteration in all worms after 24 h (**Fig. 3B**).

**Figure 4:**
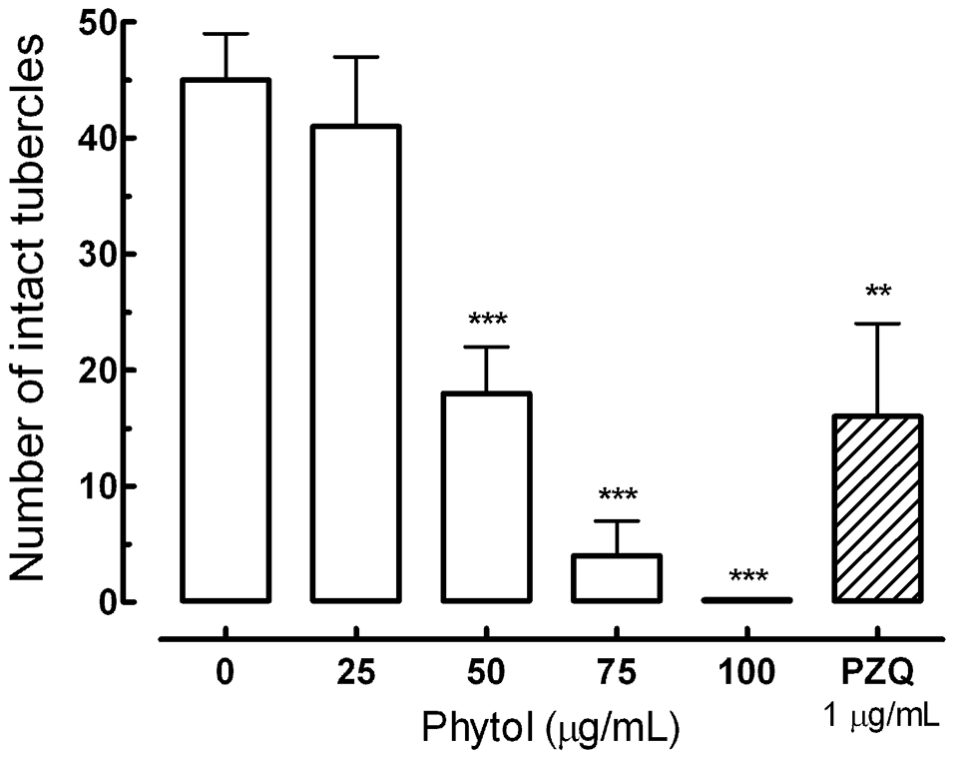
Quantitative analysis of morphological alterations on the tegument of *S. mansoni* after exposure to phytol. The quantification of the number of tubercles was performed using three-dimensional images obtained from laser scanning confocal microscopy. Indicated are numbers of intact tubercles and these numbers were measured in a 20,000 µm^2^ of area in a dorsal region of adult male worm (see Figure 2), and calculated with the Zeiss LSM Image Browser software. Praziquantel (PZQ, 1 µg/mL) was used as positive control. A minimum of three tegument areas of each parasite were assessed. Values are means ± SD (bars) of ten male adult worms. **P<0.01 and ***P<0.001 compared with untreated groups.

Additionally, morphological alterations on the tegument of *S. mansoni* were evaluated quantitatively after exposure to different concentrations of phytol. In this quantitative analysis, areas of tegument of male worms were assessed, and the number of tubercles on the dorsal surface of parasites was counted. As shown in **Fig. 4**, phytol caused changes on the tubercles of *S. mansoni* male worms in a concentration-dependent manner. For example, the number of intact tubercles in an area of 20,000 µm^2^ on male worms of the negative control group was 46, while in the group exposed to 50 µg/mL of phytol the number was 18. In addition, when the concentration of phytol was increased to 100 µg/mL, no intact tubercles were seen.

#### c. Phytol significantly reduced *S. mansoni* egg production

**Figure 5:**
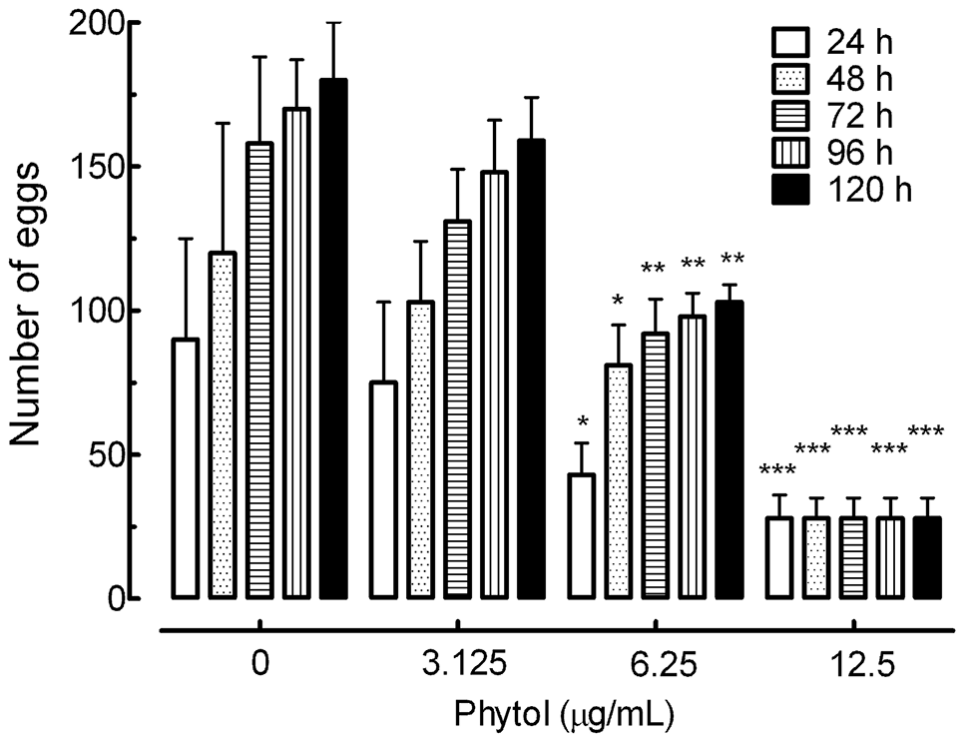
Effect of phytol on *Schistosoma mansoni* oviposition. Adult worm couples were incubated in 24-well culture plates containing RPMI 1640 medium and phytol at the indicated concentrations. At the indicated time periods, the cumulative number of eggs per worm couple was scored using an inverted microscope. Values are the means ± SD (bars) of ten worm couples. *P<0.05, **P<0.01, and ***P<0.001 compared with untreated groups.

We also evaluated the direct effect of phytol on the reproductive fitness of *S. mansoni*. In order to evaluate egg production by adult worms, samples were tested at concentrations that did not cause death or separation of all worms (phytol at concentrations ranging between 3.125 and 12.5 µg/mL; equivalent to 10.5 and 42.1 µM). As shown in **Fig. 5**, phytol significantly reduced (*P*<0.05 to *P*<0.001) the number of eggs laid by the living worms, in comparison with the number of eggs produced by the worms in the control group. At the end of the incubation period (120 h), the number of eggs was reduced by 75% with 12.5 µg/mL phytol compared with control.

To investigate whether the reduction in egg output occurred in a reversible manner, additional experiments were performed using phytol at the highest sublethal concentration (12.5 µg/mL). In this case, coupled pairs of *S. mansoni* worms were incubated with phytol for 48 h; subsequently, the medium with the drug was removed. Afterwards, the worms were incubated continuously in fresh medium without drug exposure for another 72 h. Based on three independent experiments, phytol inhibited 100% of egg laying compared with the control group. Therefore, no eggs were observed until the end of the incubation period.

### 2. *In vivo* studies

Since phytol had antischistosomal effects *in vitro*, it was further investigated *in vivo*. Therefore, the efficacy of phytol was tested against the adult parasite life stage in an experimental mammalian host. Single 40 mg/kg oral dose of phytol was administered to *S. mansoni*-infected mice at 56 days postinfection. During this period, male and female worms had matured and paired, and eggs were found in the liver, intestine, and faeces.

#### a. Oral treatment with phytol significantly reduced worm burden

**Figure 6:**
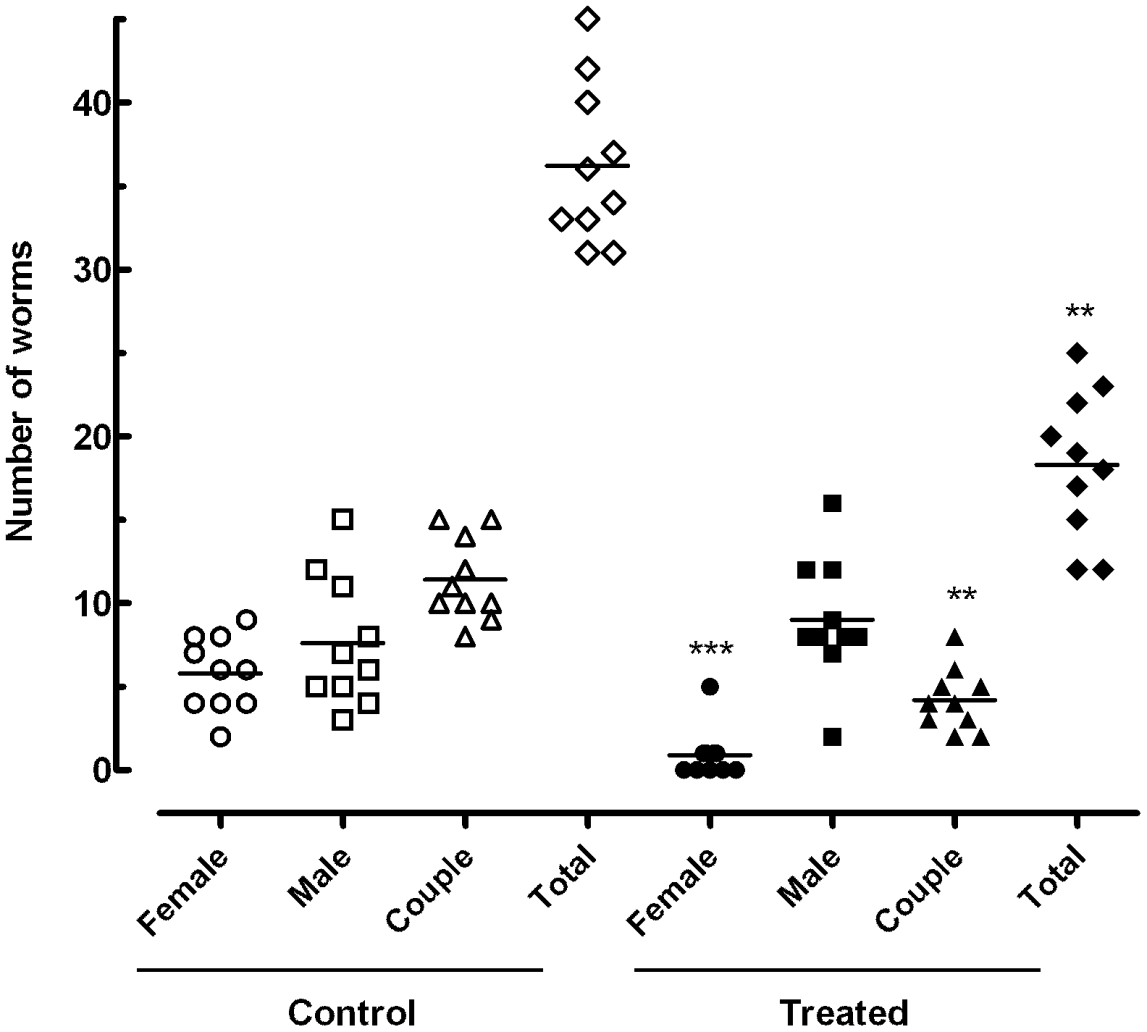
Effect on worm burden of phytol 40/kg. These results represent the effect on worm burden of a single 40/kg oral dose of phytol administered to mice harboring adult *S. mansoni* infection, stratified by sex. Drug administration started at day 56 postinfection. The animals were killed at 2 weeks post-treatment. Points represent data from individual mice that were infected and treated with phytol, or infected and untreated (control). The horizontal bars represent median values. **P<0.01 and ***P<0.001 compared with untreated groups.

In mice infected by *S. mansoni*, there was a reduction in worm burden upon oral treatment by phytol, as shown in **Fig. 6**. The treatment led to a statistically significant reduction in total and female worm burden compared with infected untreated controls (*P*<0.01), with a reduction of 51.2% and 70.3%, respectively. The number of worm couples was also considerably reduced compared with the control infections (*P*<0.01), with a reduction of 63.2%. Moreover, highly significant unpaired female worm burden reductions (83.7 to 100%) were achieved in the mouse model compared with infected untreated controls (*P*<0.001). In contrast, there were no significant differences in the total number of unpaired male worms compared with infected untreated controls. Similar results were obtained in the second set experiments using adult parasite stages (**Fig. S1**).

#### b. Oral treatment with phytol significantly reduces egg production

**Figure 7:**
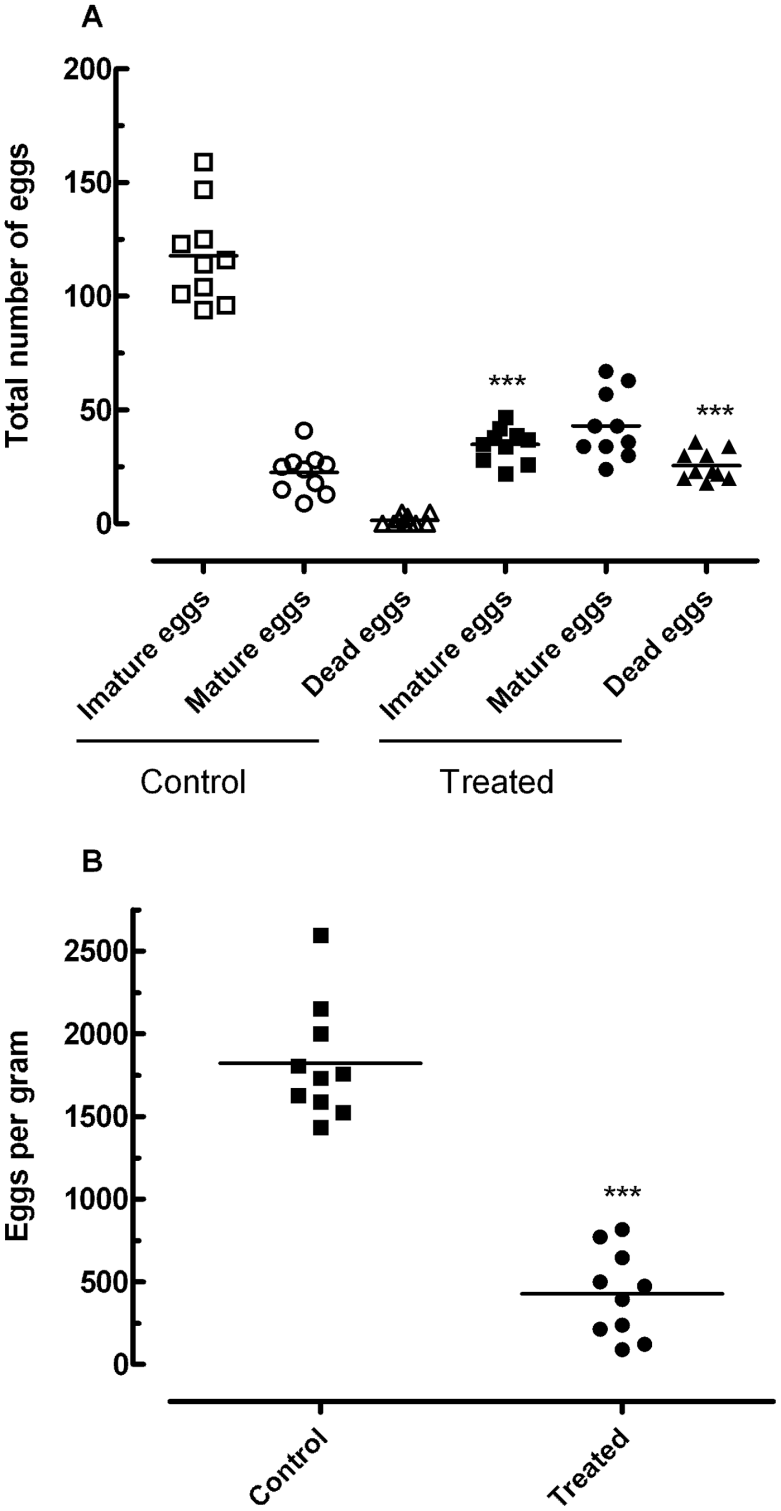
Effect on egg development stages (A), and stool egg load (B) of phytol 40 mg/kg. These results represent the effect on egg development stages (oogram pattern), and stool egg load of a single 40 mg/kg oral dose of phytol administered to mice harboring adult *S. mansoni* infection. Drug administration started at day 56 postinfection. The animals were killed at 2 weeks post-treatment. Points represent data from individual mice that were infected and treated with phytol, or infected and untreated (control). The horizontal bars represent median values. ***P<0.001 compared with untreated groups.

The effect of phytol on egg development stages (oogram pattern) and faeces egg load at day 56 postinfection is shown in **Fig. 7**. In the wall of the intestine, eggs of all developmental stages were observed in the treated group, but the frequency of immature eggs was significantly lower compared with infected untreated controls (*P*<0.001). The oogram also showed significant increases in the proportion of dead eggs compared with the control group (*P*<0.001) (**Fig. 7A**). In faeces samples collected from mice, the Kato-Katz method revealed that phytol significantly reduced the number of eggs compared with the infected untreated control group (*P*<0.001), with a reduction of 76.6% (**Fig. 7B**).

#### c. Tegumental changes in adult *S. mansoni* harboured in mice treated with phytol

**Figure 8:**
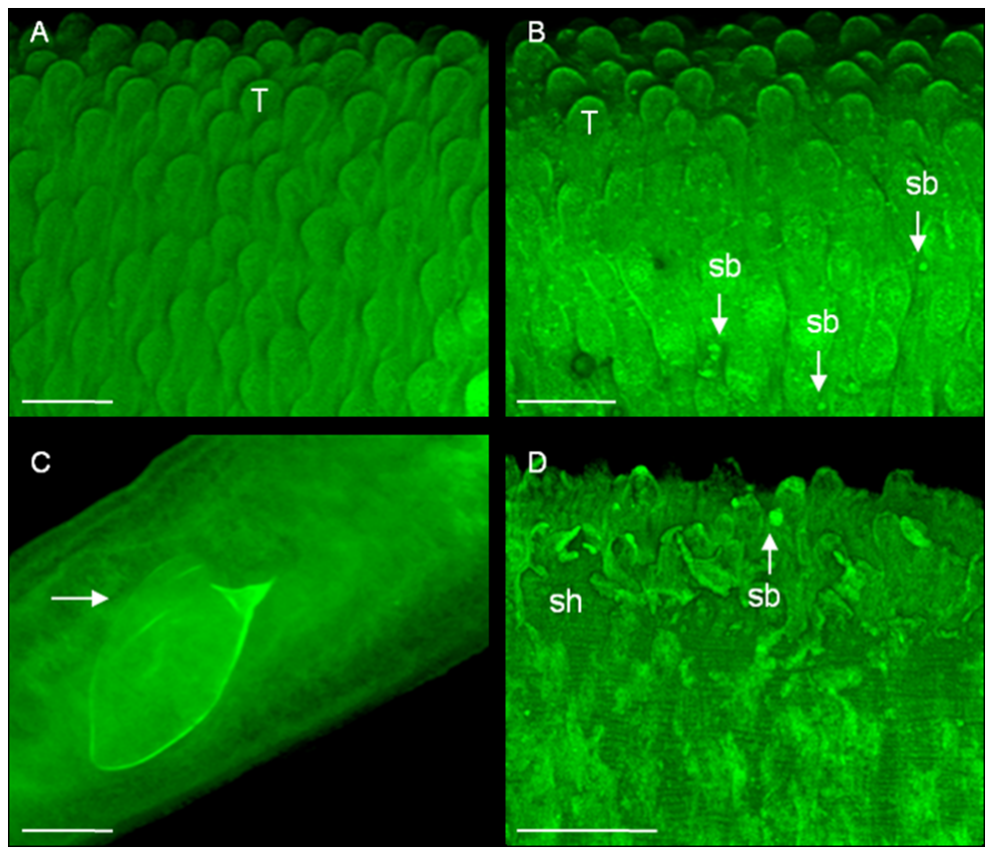
Microscopy observations of adult *Schistosoma mansoni* recovered from mice. Animals were treated with a single oral dose of phytol (40 mg/kg) at day 56 postinfection. The animals were killed at 48 h post-treatment and fluorescent images were obtained using confocal laser scanning microscopy. **A:** Male worms from infected and non-treated animal (control); dorsal tegumental surface showing tubercles (T). **B:** male worms from infected animals and treated with phytol; dorsal tegumental surface showing blebs (sb), **C:** female worms from infected and non-treated animal (control); female schistosome showing an intra-uterine egg (arrow). **D:** female worms from infected animals and treated with phytol; tegumental surface showing blebs (sb) and extensive sloughing (sh). Scale bars = 50 µm.

The results of confocal laser scanning microscopy of male and female *S. mansoni* recovered from infected untreated mice (Group IIIa) and phytol-treated mice (Group IIIb) for 48 h are presented in **Fig. 8**. After phytol treatment, male schistosomes did not show morphological alterations in tegumental tubercles, although there were small blebs emerging from the tegument around the tubercles (**Fig. 8A**) compared with the infected untreated controls (**Fig. 8B**). In contrast, there was apparent damage to the tegumental surface of female schistosomes, with extensive sloughing, and the tegument appeared to be swollen (**Fig. 8C**) compared with untreated controls (**Fig. 8D**). Phytol alters the tegument of both paired females and unpaired females, but the damage is more noticeable in unpaired females.

## Discussion

Phytol is widespread in nature, especially because it occurs ubiquitously as a component of chlorophyll [9]. It is considered a common food additive and information about oral bioavailability of phytol in mice revealed that this drug is well absorbed (30–66% of the administered dose) [32]. Moreover, comprehensive toxicological data are available. For example, the acute oral LD_50_ of phytol in rats was reported to be greater than 10,000 mg/kg and it was also not considered mutagenic [10].

This study has highlighted *S. mansoni* as a possible new target for phytol. Initially, we examined its antischistosomal activity on adult worms *in vitro* and the results encouraged us to examine its efficacy in mice harbouring adult *S. mansoni*. *In vitro* assays demonstrated that phytol affected parasite motility, viability, and egg production and it induced severe tegumental damage in schistosomes. Additionally, various parasitological criteria indicated the *in vivo* antischistosomal effects of phytol: it caused significant reductions in worm load, faeces egg load, and the frequency of egg developmental stages. To the best of our knowledge, we have, for the first time, evaluated the activity of phytol against the laboratory model *S. mansoni in vitro* and *in vivo*.

In general, our *in vitro* experiments on adult schistosomes confirmed the promising *in vivo* results. Indeed, the *in vitro* bioassay revealed that phytol acted preferentially against female rather than male worms. Likewise, the effect on worm burden of a single 40 mg/kg oral dose of phytol administered to mice harbouring a 56-day-old adult *S. mansoni* infection clearly showed that females were more susceptible than males. A similar variation in drug susceptibility between male and female schistosomes both *in vitro* and *in vivo*, has been observed with several antischistosomal drugs. For example, Mitsui et al., 2009 [33] reported that female worms of *S. mansoni* were often more susceptible than males to artenusate *in vitro*. Keiser et al. (2009) [34] described that female adult worms were more affected by mefloquine than male adults when the drug was administered orally to mice infected with adult *S. mansoni*. A similar finding was previously reported by Botros et al. (2003) [35] when testing the activity of the acyclic nucleotide analogue 9-(S)-[3-hydroxy-2-(phosphonomethoxy)propyl]adenine [(S)-HPMPA] against experimental schistosomiasis mansoni.

Unlike other recently described schistosomicides such as the cysteine protease inhibitor K11777 [36] or the oxadiazoles [37], which thus far have only been tested intraperitoneally and in multiple doses, phytol at a single oral dose resulted in worm burden reductions. Although our results were moderate with respect to total worm burden reduction (51.2%), high female worm burden reductions (70.3%) were observed in *S. mansoni*-infected mice treated with phytol. Besides, in contrast to the effects of the recognised antischistosomal drug praziquantel, which kills adult schistosomes (male and female), the killing effect of phytol is weaker. In this respect, *in vitro*, all adult worm pairs were separated into individual male and female worms after 24 h of incubation with phytol at concentrations of 25 µg/mL and above. Male and female worms were unable to embrace and mate and remained separated, and 100% of the female and male worms were dead after 24 h of incubation with phytol at concentrations of 50 and 100 µg/mL. A single oral dose of phytol administered to mice did not cause a significant reduction in the load of unpaired male worms. Nevertheless, treatment with phytol resulted in the death of most of the *S. mansoni* unpaired females (no unpaired female was recovered from five mice; one unpaired female was recovered from four mice; and four unpaired females were recovered from one mouse). The reduction of coupled worms and total load of worms may be due to the decrease in the number of female schistosomes.

Since the tegument of schistosomes is an important target for antischistosomal drugs, alterations in the surface topography of schistosome worms were used by several investigators for the evaluation of antischistosomal drug activities *in vitro* and *in vivo* [e.g. 25], [38]–[42]. In our *in vitro* assays, confocal laser scanning microscopy revealed that phytol induced severe tegumental damage in both male and female schistosomes. Additionally, quantitative analysis showed that phytol caused changes on the tubercles of *S. mansoni* male worms in a dose-dependent manner. Comparable results were obtained by previous works using other antischistosomal compounds, such as piplartine [22], dermaseptin [26], and (+)-limonene epoxide [43]. Based on our *in vitro* analysis, the anatomical disturbance differed between the reference drug (praziquantel) and phytol. Praziquantel caused severe muscle contractions and the worms became partially curved or swirled. In contrast, phytol caused worm paralysis but not muscle contraction.

Also, the present observations of alteration in the surface architecture of *S. mansoni* male worms as a result of treatment with phytol are not similar to the tegumental alterations seen *in vitro*. In this sense, at the confocal microscopic level, the male parasites recovered from mice 48 h after phytol treatment did not show significant morphological alterations, although blebbing was visible on the tegument of male worms. Blebbing is an indicator of stress and has been observed in previous studies evaluating antischistosomal drugs such as carvacryl acetate [23], mefloquine [38], miltefosine [39], artesunate and praziquantel [44], [45]. The discrepancy between the effect of the drug and the onset of action of phytol *in vitro* and *in vivo* might be related to the lower phytol concentrations present in the liver and mesenteric veins in mice compared with the *in vitro* model. Moreover, these differences between *in vitro* and *in vivo* results may be explained by the fact that *in vitro*, the parasite is in a direct contact with the drug and, thus, it is not in direct contact with the host's microenvironment. Pharmacokinetic studies, measuring drug concentrations in the body and the target organs, might aid in the elucidation of these differences observed *in vitro* and *in vivo*.

Furthermore, in contrast to adult male schistosomes, which have some blebs on the tegument, a single oral dose of 40 mg/kg phytol resulted in extensive tegumental damage of female worms. These results confirm, at least partially, that phytol is orally bioavailable [10], but possibly the oral dose must be increased to achieve tegumental damage in male worms. We speculate that there is either sex-specific interference of the drug with the target, or that there are different targets for phytol in females compared with males. Nonetheless, further research is needed to provide a better understanding of the schistosomicidal action of phytol.

Tegumental damage may not always result in death [46], but the morphological alterations observed in this study could be a mechanism through which phytol kills the worms. The damage to the tegument along the worm's body would have impaired the functioning of the tegument and also destroyed the defence system of the worm, and so it could easily be attacked by the host's immune system. Further studies are necessary to elucidate the multiple mechanisms of action of phytol, which seem to be involved in the killing of schistosomes. It might also be useful to investigate whether phytol acts synergistically with the host immune response, similar to the chemotherapeutic effect of praziquantel, which has been shown to be dependent on the host antibody response [47], [48]. On the other hand, it is possible that phytol has a direct killing effect without the absolute need for antibody, which occurs with praziquantel [49].

Finally, to see whether phytol affects the sexual fitness of adult worms, we evaluated the number of eggs *in vitro* and development stages (oogram pattern) and faeces egg load in *S. mansoni*-infected mice. Reductions in worm recovery and egg density in treated mice and *in vitro* were observed; this is considered by several authors as strong evidence of the efficiency of antischistosomal drugs [e.g.], [ 19], [39], [40], [42], [50,51]. The reduction of egg load in the tissues and faeces in treated mice may be attributed to the reduction in worm burden as a result of phytol treatment, the low productivity of the female already present, and the active destruction by the host's tissue reaction of the few eggs produced. *In vitro*, phytol caused a 75% reduction in egg production compared with untreated worms, although it is known that *in vitro* egg production is spontaneously reduced after a few days in culture [24]. However, more importantly, the inhibition of oviposition was irreversible, as found by examination of the worms following washing and incubation in drug-free RPMI medium, whose effect has been reported in previous studies with other antischistosomal compounds such as epiisopiloturine [25], piplartine and dermaseptin [24]. *In vivo*, significant alterations in oogram patterns and faeces egg load were found and, thus, phytol affected the fecundity of the worms and/or the viability of the eggs. As described by Sanderson et al. (2002) [52] for *in vitro* and *in vivo* studies on the bioactivity of a ginger extract, it is not known whether these anti-fecundity effects were the result of generalised cytotoxic damage or more specific inhibition of reproductive process by phytol. It is known, furthermore, that inhibitors of cholesterol synthesis such as lovastatin (mevinolin) can reduce egg laying by *S. mansoni* females. For example, Vanderwaa et al. (1989) [53] has demonstrated with lovastatin that egg production by *S. mansoni*, *in vitro* and *in vivo*, is associated with the enzyme hydroxymethylglutaryl-coenzyme A (HMG-CoA) reductase, and that cholesterol precursors, mevalonate and farnesol, stimulate egg production by the female parasite and can reverse mevinolin-induced inhibition of egg production. Subsequently, Chen et al. (1990) [54] demonstrated that mevalonate and/or its metabolite not only plays a vital role in schistosome egg production but is also vital for survival of the parasite.

Importantly, recent investigations have demonstrated that phytol is a cholesterol-lowering agent [55]. Accordingly, we speculated that the reduction in oviposition by phytol may be associated with the inhibition of HMG-CoA reductase. However, the underlying mechanism(s) of the effects remains to be fully elucidated. Presumably, it may also be due to a direct assault on female worms, thus diminishing their numbers or their ability to lay eggs, although a direct ovicidal action cannot be excluded [35]. Results obtained from preliminary morphological investigations (no data shown) indicate that phytol exerts a rapid action on schistosomes, resulting in marked alterations of the reproductive system of the worms.

The main targets of the action of phytol are the female worms in terms of either the load of female worms or their ability to lay eggs. Egg production is contingent on worm maturation, pairing, and the support of the metabolic needs of the female. Phytol clearly disrupted this development process by directly killing female worms or inhibition of oviposition. In schistosomiasis, reductions in worm burden are associated with reduced pathology, and there is no concern about relapse because schistosome parasites do not multiply in the mammalian host. Moreover, reduction in egg burden can reduce egg shedding and the potential for parasite and disease propagation.

In conclusion, the present results suggest that phytol has antischistosomal activities and provide a basis for subsequent experimental and clinical trials. The low toxicity and high bioactivity and tolerance by mammals support the potential of phytol as a new lead compound for human schistosomiasis. However, the effect of phytol in both *in vitro* and *in vivo* studies was evaluated using *S. mansoni* adult worms; thus, further studies are needed to evaluate the efficacy of phytol in different therapeutic regimens (e.g., multiple oral doses) and to evaluate the efficacy of this drug against different life-cycle stages (e.g., schistosomula and juvenile worms) as well as other *Schistosoma* species. Additionally, the detailed mechanism of action of phytol on schistosomes remains to be investigated.

## Supporting Information

Alternative Language Abstract S1 Translation of the Abstract into Portuguese by Josué de Moraes.
(DOC)

Figure S1 Effect on worm burden of phytol 40 mg/kg (second set of experiment). These results represent the effect on worm burden of a single 40 mg/kg oral dose of phytol administered to mice harboring adult *S. mansoni* infection, stratified by sex. Drug administration started at day 49 postinfection. The animals were killed at 1 week post-treatment. Points represent data from individual mice that were infected and treated with phytol, or infected and untreated (control). The horizontal bars represent median values. **P<0.01 and ***P<0.001 compared with untreated groups.
(TIF)
