# Supplementary material for: Phytol, a Diterpene Alcohol from Chlorophyll, as a Drug against Neglected Tropical Disease Schistosomiasis Mansoni
Source: PLoS Negl Trop Dis. 2014 Jan 2;8(1):e2617. doi: 10.1371/journal.pntd.0002617 (PMC3879229; doi:10.1371/journal.pntd.0002617)
Supplement: Alternative Language Abstract S1 — Translation of the Abstract into Portuguese by Josué de Moraes. (DOC) [file pntd.0002617.s001.doc]

**Fitol, um diterpeno álcool da clorofila, como uma droga contra a esquistossomose mansônica**

Translation of the Abstract into Portuguese by Josué de Moraes

**Resumo**

**Introdução:** A esquistossomose é uma doença parasitária negligenciada e continua sendo um dos principais problemas mundiais de saúde pública. O tratamento dessa doença é baseado apenas na quimioterapia com o praziquantel e, portanto, é premente a necessidade de novas alternativas terapêuticas. Neste estudo é relatado que o fitol, um diterpeno álcool da clorofila comumente usado como aditivo alimentar, possui efeito anti-helmíntico *in vitro* e *in vivo* em *Schistosoma mansoni*.

**Metodologia/Principais Resultados:** Estudos *in vitro* mostraram que o fitol (50 µg/mL a 100 µg/mL) reduziu a motilidade dos vermes adultos de *S. mansoni* e foi letal para os parasitas. Além disso, análise por microscopia confocal revelou alterações no tegumento dos helmintos de maneira dose-dependente. Nas concentrações subletais do fitol (25 µg/mL) notou-se redução na oviposição dos parasitas. Em animais, dose única de fitol, 40 mg/kg via oral, reduziu a carga parasitária total e das fêmeas de *S. mansoni* em 51,2% e 70,3%, respectivamente. Além disso, o fitol significativamente reduziu o número de ovos nas fezes (76.6%) e o oograma revelou uma queda na quantidade de ovos imaturos e aumento de ovos mortos. Alterações tegumentares foram vistas, por microscopia confocal, em vermes adultos recuperados de animais tratados com fitol, sobretudo nas fêmeas.

**Conclusões e Importância:** A redução significativa da carga parasitária após o tratamento com o fitol mostra que esta molécula é um agente antiparasitário promissor contra a esquistossomose. Fitol é um aditivo alimentar seguro, pois não apresenta toxicidade ou mutagenicidade, e de baixo custo. Portanto, fitol é um importante candidato como fármaco para o tratamento da esquistossomose.
